# Public Awareness of Remanufactured Products in Yangtze River Delta of China: Present Status, Problems and Recommendations

**DOI:** 10.3390/ijerph15061199

**Published:** 2018-06-07

**Authors:** Jian Cao, Xihui Chen, Xueping Zhang, Yanchen Gao, Xuemei Zhang, Yunwen Zhao, Xiaoli Yang, Jiayang Xu, Gengui Zhou, Jerald L. Schnoor

**Affiliations:** 1 Department of Management Science & Engineering, Zhejiang University of Technology, Hangzhou 310023, China; jcao@zjut.edu.cn (J.C.); jerrychen0526@foxmail.com (X.C.); xpz1993@hotmail.com (X.Z.); gyc511@foxmail.com (Y.G.); zhaoyw0601@foxmail.com (Y.Z.); yxl_33793@163.com (X.Y.); jiayangxu@zjut.edu.cn (J.X.); ggzhou@zjut.edu.cn (G.Z.); 2 Center for Global & Regional Environmental Research, The University of Iowa, Iowa City, LA 52242, USA; jschnoor@engineering.uiowa.edu; 3 Department of Civil and Environmental Engineering, The University of Iowa, Iowa City, LA 52242, USA

**Keywords:** remanufactured products, used products, questionnaire survey, public awareness

## Abstract

Exponential increase of used and scrapped products has aroused worldwide attention, with various coping strategies regarding environmental protection and resource reutilization being considered and implemented. Among these, remanufacturing, processing used products environmentally and restoring them to like-new conditions, is preferred by nations around the world. China has been committed to developing and advancing the remanufacturing industry along with its products since 2013, however only a few residents are able to recognize and purchase remanufactured products at the present time. This paper aims to investigate the public awareness of Chinese residents on these emerging products by conducting a questionnaire survey and field research for data collection, and analyzing the results statistically. Results show that most Chinese residents are not familiar with remanufactured products, the superior attributes of such products, or the channels available to purchase them. This could be explained by insufficient publicity and promotion on the part of the government and business enterprises. Factors influencing the purchase intentions of customers are illuminated and potential problems are summarized, in response to which, respective recommendations are provided for both policy makers and firms to popularize remanufactured products.

## 1. Introduction

Owing to technology innovation and mass industrial production, new products have burgeoned around the world, generating many waste products, and severely affecting the environment [1,2,3,4,5]. Confronting the situation, remanufacturing, which involves a series of processes that restore end-of-life products, parts or components, and returns them back to as-new conditions, has become a global strategy to deal with the massive amount of scrapped products [6,7,8,9]. The scrapped products processed by remanufacturing are referred as “Remanufactured Products” which can match the same properties and performance of the original ones [10,11,12].

Compared with the newly made products, remanufactured items should be more favored for their attributes of energy-savings, less material-consumption and lower emissions [13]. Unlike conventional manufacturing that aggravates resource depletion and environmental pollution, remanufacturing has an edge in saving ~60% electrical energy, reducing ~70% consumption of metallic materials and decreasing ~80% emissions of air pollutants [14,15]. Taking a combustion engine for instance, producing one remanufactured engine, other than a new one, can decrease emissions by 565 kg CO_2_, 6.09 kg CO, and 3.98 kg SO_2_ [16]. These enhanced resource re-utilization and environment benefits of remanufacturing can be supplemented by improved economic utility. The retail price of remanufactured products in China, for example, is 70–80% of the original [17,18]. Such competitive pricing of remanufactured commodities indicates their potential to be preferred by customers.

China has devoted itself to recycling for a long time, while the remanufacturing industry did not enter into its rapid development until four years ago with the publication of two essential policies, *Swap the Old for Remanufacturing* in 2013 and *Remanufacturing Sample Pilot Enterprises* in 2014 [19,20]. Implementation of the former stipulation helps remanufacturing enterprises get subsidies of different types for used products from the government directly. The latter has selected 76 pilot remanufacturing enterprises from 2014 to 2016 to guide medium- and small-sized enterprises in the same or related sectors, and to support them technologically [21,22]. Pilot remanufacturing enterprises such as SCMRC (Sany Construction Machinery Remanufacturing), XCMG (Xuzhou Construction Machinery Group) and WRG (Weichai Remanufacturing Group) selected into the lists of *Remanufacturing Sample Pilot Enterprises* have received much financial and technical assistance from the government [23,24,25]. Inspired by the two policies, remanufactured products in this way have begun to prosper. According to *The List of Remanufactured Products* published by Ministry of Industry and Information Technology (MIIT), more than 10,000 types of remanufactured products with domestic certifications were qualified for sale by the end of 2016 [26].

Research in the field of remanufacturing industry is gradually becoming one hot-spot, which is conducted from both modeling and empirical points. Savaskan et al. [27] investigated collection rates of used products in different models in a closed-supply chain via game theory. Chen [28] used agent-based simulation and evolutionary game theory in the light of innovative diffusion process with network externality and sustainability consciousness in dynamic emergent perspectives. These studies above were conducted via mathematical modeling. Some scholars utilized empirical method to investigate the relations between remanufactured goods with customers. Hamzaoui-Essoussi et al. [29] investigated the impacts of product category and perceived risk on consumers’ willingness to pay for recycled/remanufactured products. Agrawal et al. [30] used a series of behavioral experiments to identify how the remanufacturers influence the perceived value of new products. Khor et al. [31] adopted planned behavior theory and utilized relevant data of Malaysia to analyze consumer attitude and subjective norms of remanufactured products. Matsumoto et al. [32] made comparisons of consumers’ perceptions of remanufactured auto parts in the United States and Japan. Watson [33] figured out public attitudes, perceptions and behavior which relate to remanufactured, repaired and reused products, and concluded price of the commodities is a significant factor. Wei et al. [34] distributed questionnaires to research on motives and barriers of remanufacturing in China from the perspective of enterprises. Wang et al. [35] indicated that purchase intention to remanufactured products is directly influenced by purchase attitude followed by perceived behavioral control and indirectly influenced by perceived risk, perceived benefit and product knowledge. Wang et al. [36] utilized innovation diffusion and customer perceived value theory to investigate consumer value considerations in the closed-loop supply chains.

Observations of previous studies on remanufacturing show that existing studies of remanufacturing mainly focus on mathematical modeling and empirical studies which may underestimate the role of public involvement in remanufacturing industry. Specific contribution of the current paper is to design applicable methods of questionnaire survey, field research, interview, etc. to investigate public awareness of Chinese residents on remanufactured products. Compared with previous studies, our research focused on the perspective of consumers and found the information and purchase channels of remanufactured products for the public customers. We analyzed different factors such as the gender and age that impact customers’ purchase intentions of remanufactured commodities. Various pre-purchase considerations of customers were also investigated. Results and findings show that public awareness is important for popularizing remanufactured products. Some of our findings verify previous research results in the field of remanufacturing while other findings that are different from previous studies have also been put forward. Our research, based on the perspective of consumers, is distinguishable from previous studies that focuse on government regulations or enterprise operations, thus will provide adaptable measures to propagate the remanufactured products.

As one type of emerging and innovative products, remanufactured products are expected to be recognized and preferred by the public, with the prospect of further promotion [37,38,39]. Only when residents have the basic knowledge of the remanufactured product are they willing to purchase it, and the government as well as remanufacturing enterprises can further disseminate it [40,41]. This paper aims to investigate the extant status of public awareness of remanufactured products, with the Yangtze River Delta selected as a typical region in China for a questionnaire survey. In Section 2, materials and methods are introduced to show the design idea of the raw material collection, the procedure of data processing and analysis. Statistical results are demonstrated and discussed in Section 3. Section 4 illustrates existing problems based on the materials collected, with respective suggestions put forward for the government and remanufacturing enterprises. Section 5 concludes the results with managerial implications and orientations for future research.

## 2. Materials and Methods

The current paper is oriented to decipher the public awareness of Chinese residents on remanufactured products. To achieve such an objective, our research team utilized a series of methods to collect raw data and related materials. The methods include: (1) questionnaire design and distribution; and (2) field research and interview. In particular, since statistical results of data analysis are important to both remanufacturing enterprises and the government to popularize the remanufactured products in China, we utilized extensive data analysis method to guarantee the validity and rationality of the results. After recycling questionnaires and collecting raw data, our research team used SPSS software to process the validity of questionnaires. Furthermore, cross-impact and descriptive statistics were adopted to analyze the valid data to get reliable results and beneficial implications.

### 2.1. Raw Data Collection

#### 2.1.1. Questionnaire Design and Distribution

**Figure 1:**
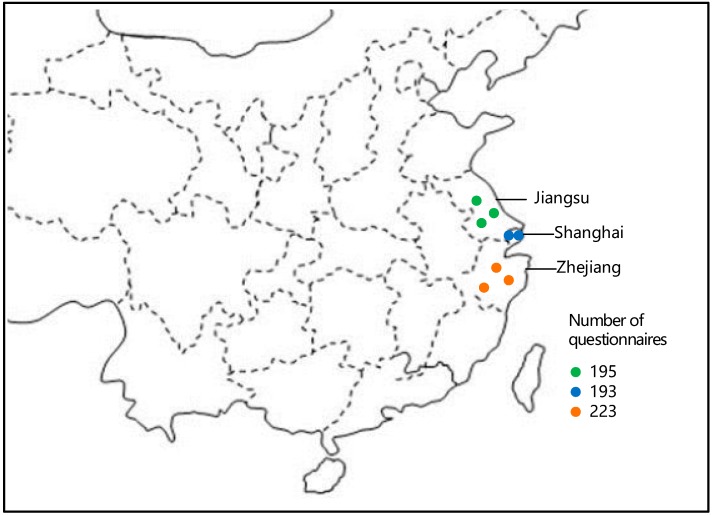
The geographical distribution of the questionnaires.

In this survey, questionnaires were allocated to collect raw data in the Yangtze River Delta, which is one of the largest delta areas in China comprised of Zhejiang Province, Jiangsu Province, Shanghai City, etc. The Yangtze River Delta covers 210,000 km^2^, which is close to the territory area of the United Kingdom, and the population in this area is up to 160 million which is nearly half the population of the USA (320 million). Due to the high density of population and prosperous manufacturing industry in this region, more than 80% pilot enterprises of remanufacturing in China were established here. Yangtze River Delta is therefore frequently regarded as a typical area for empirical studies in the field of remanufacturing industry by questionnaires. During about five months of investigation, we distributed 700 questionnaires and received 611 valid ones, with a response rate of 87.3% eventually by the end of September 2017. Figure 1 shows the distribution of questionnaires. 

Our research team spent more than 20 days in each city of Zhejiang and Jiangsu Provinces to distribute questionnaires in campuses, markets, public squares, etc. In the capitals of Zhejiang and Jiangsu Province, we collected 16.2% and 15.8% of questionnaires in Hangzhou and Nanjing, respectively. Shanghai, one of largest municipalities in China, provided 13.5% questionnaires for our research team. To further improve the validity of questionnaire samples, we also distributed questionnaires in other small- and medium-sized cities. In Ningbo, Taizhou and Huzhou of Zhejiang Province, for instance, we collected 11.8%, 9.4% and 8.8% of total questionnaires, respectively. Overall, 9.5%, 8.2% and 7.8% of valid questionnaires were retrieved in Wuxi, Suzhou and Changzhou of Jiangsu Province, respectively.

To deepen the understanding of the questionnaire for respondents, the original questionnaire was designed in Chinese. During questionnaire distribution, our research team would explain the details about the questions and the options. The questionnaire was then translated into English, which is the Appendix A of this paper. We classified the questionnaire consisting of 21 questions into five groups, after structure division, as follows.

Group 1 (Questions 1–6) was designed to get the basic information of the respondents, such as their gender, age, and education level. Ages of the respondents ranged from 12 to 74, and generally form a logical distribution of age. The age distribution of the survey population is basically consistent with the age structure of population in China which was published by NBSC (National Bureau of Statistics of China) in 2017 [42]. For instance, 13.3% of respondents in our survey are under twenty years old, while the portion of the same age published by NBSC is 13.1%. In total, 17.7% of respondents were in their thirties (30–39); this portion in national population occupies 16.1%. In our survey, 20–29-year-old respondents represented 41.1%; 22.1% of respondents were 40–49 years old; and 5.9% were older than 50 years. The demographic results are shown in Table 1.

Group 2 (Questions 7–10) was aimed to investigate the promulgation of promotional channels of the remanufactured products. For example, we asked the following questions: *Have you ever purchased remanufactured products? Which channels help you know the remanufactured products? Which channels that promulgate the remanufactured products do you trust most?* By posing such questions affixed with multiple answer choices, general ideas and preferences for the promotion channels of Chinese residents possessed with primary understanding of remanufactured products were attained.

Group 3 (Questions 11–14) was to understand the pre-purchase considerations that may affect customers’ purchase intentions. Take Question 14, for instance: *In which respects do you care most about the remanufactured products?* Multiple selections of 10 items were attached to this question, such as Performance, Quality and Certification of the remanufacturing enterprises, which were essential to understanding pre-purchase considerations in detail. Based on the detailed results of the pre-purchase considerations, the corresponding suggestions were recommended for the government and enterprises to put forward prudent policies and to make sensible marketing strategies, respectively.

Group 4 (Questions 15–18) described the public awareness on purchase channels of remanufactured products, such as Question 16: *Do you know where you can purchase the remanufactured products?* Several options such as *Sales network of the original manufacturers* and *Electronic business* were provided. In practice, there are various channels in the Chinese remanufacturing market through which customers can purchase remanufactured commodities. Questions in Group 4 were distributed to figure out the recognition level of residents on these channels. By fulfilling such an assignment, the most influential channels could be strengthened and underscored, and new sales networks could be developed and recommended for enterprises.

Group 5 (Questions 19–21) was posed to investigate the public awareness of the necessity to popularize the remanufactured products in China as well as the responsibility for environmental protection. For example, Questions 19 and 20: *Do you think it is necessary to popularize the remanufactured products? Who do you think should be responsible for popularizing the remanufactured products?*

#### 2.1.2. Field Research and Interviews

In addition to questionnaire survey, field research was also conducted via interviews to document awareness of certain people on remanufactured products. During the field research, our research team visited campuses, shops, squares and other public places in each surveyed city to interview people who have different occupations such as teachers, salesmen and managers in the shops, students, etc.

Some example questions of interview are introduced as follows:

- Have you ever heard of remanufactured products? Do you know the concepts of it?
- Which attributes of remanufactured products do you care most? For example: price, capacity etc.
- Which information channels help you to understand remanufactured products?
- Have you ever heard some national policies related to remanufacturing such as ‘Swap the Old for Remanufacturing’?
- Who do you think should undertake the responsibility of environmental protection?

All answers to these interview questions were recorded in documents. Through such endeavor, field research and interview provide us extra materials to verity the outcomes of questionnaires.

### 2.2. Data Analysis Method

#### 2.2.1. Raw Data Processing

We manipulate the raw data in questionnaires carefully to guarantee the reliability of data. The procedures of raw data processing are introduced as follows

(1) Check the authenticity of answers in questionnaire by observing the questions which are familiar or opposite. For instance, if Question A is familiar to Question B, some respondents replied “*Yes*” in Question A but give answer “*No*” to Question B. Their answers in questionnaires will be queried (SPSS software has the function of data verification). 

Taking our questionnaire for the typical case, if the respondent selected the option “*The qualities of remanufactured products are not as same as the new products*” in Question 12, but did not select “*Quality*” in Question 14, such invalid answers will be screened out.

(2) Check the repetition rates of answers. If in some questionnaires, several questions are all answered via similar options, these questionnaires will be regarded as invalid ones (SPSS software has the function of repetition rates checking).
(3) Check the questions that are not answered by respondents. To guarantee the rationality of questionnaire research, if 15% of questions in one questionnaire are not answered or have the answer “*Unsure*”, it will be regarded as invalid one.

After the manipulation of raw data, our research team obtained 611 valid questionnaires whose results can be analyzed further. All the manipulation procedures are conducted via SPSS software.

#### 2.2.2. Cross-Impact Analysis

After raw data collection, we processed the data by cross-impact analysis method to obtain the scientific results and conclusions.

Cross-impact analysis method is a standard tool for the scenario prediction based on enough samples [43]. It provides several structured processes for the deduction of plausible developments of the future [44]. The benefits of cross-impact analysis can be classified into four points.

(1) Cross-impact analysis method can process multiple variables to outline the correlation between them directly.
(2) Cross-impact analysis method can help the analyst, forecasters and readers to explore how certain factors in the questions are likely to interact with each other.
(3) Cross-impact method provides a conceptual structure into which data can be suited, so that the rationality and interaction of data can be tested subsequently.
(4) Cross-impact method has a profound range of application, such as capital requirements, environmental impact, job creation, etc. Our research team adopted it to measure the public awareness on remanufactured products.

The data collected were processed based on probability theory principles and following mathematical equations.

(1) $$P_{i}=\frac{N_{i}}{N_{total}}$$

(2) $$P_{\left(i\cap j\right)}=\frac{N_{\left(i\cap j\right)}}{N_{total}}$$

(3) $$Impact\left(i,j\right)=\frac{P_{\left(i\cap j\right)}}{P_{i}}$$

In Equations (1)–(3), *i* and *j* signify the options that respondents have selected. $N_{i}$ is the number of people who choose option *i*, therefore $P_{i}$ is the probability of $N_{i}$ to $N_{total}$. $N_{\left(i\cap j\right)}$ means the population who selected option *i* and *j*, and $P_{\left(i\cap j\right)}$ is the corresponding proportion. Impact(i,j) indicate the influence degree of $P_{\left(i\cap j\right)}$ to $P_{i}$.

Due to the development of statistical analysis software, cross-impact analysis method is regarded as a powerful tool to process a series of data. In this paper, we utilized SPSS 19.0 to process the raw data in cross-impact method.

#### 2.2.3. Descriptive Statistics Analysis

Survey research can be divided into three classes: exploratory, confirmatory and descriptive [44,45]. The exploratory research aims to put forward and define a new research issue, while confirmatory is to test a theory. The Chinese remanufacturing industry has been developing for more than ten years, with dramatic contribution made in the research field. Hence, the utilization of exploratory survey is unrealistic for us to define related issues. Besides, since few studies have been conducted on public awareness of remanufactured products in China, it will be difficult to carry out confirmatory research to verify hypotheses that have been put forward in existing literature. 

Unlike exploratory and confirmatory survey method, descriptive statistics is acknowledged as the applicable path to comprehend the relevance of a phenomenon and outline the incidence or the distribution in a population. As the most basic type of enquiry, descriptive statistics method aims to examine a situation via multiple factors and produce many data in a short time with a fairly low cost [46,47]. Descriptive survey method as an independent survey approach is mainly described as a method for identifying, analyzing and reporting patterns with data [48]. 

Hence, we adopted descriptive statistics to investigate the current situation about public awareness or attitudes toward remanufactured products. To present the public awareness of remanufactured products in a most intuitive and graphical way, this paper utilized descriptive statistics in addition to exploratory or confirmatory method to display the data analysis results of valid questionnaires. 

## 3. Results and Discussion

The remanufacturing industry has been expanding since 2013 in Mainland China, and the government published a series of regulations and policies to promote this emerging industry. Remanufacturing enterprises have been offered much direct support in capital and technology [48,49,50,51]. Notwithstanding its many positive prospects, the remanufacturing industry in China could come to a halt due to the relatively deficient knowledge of customers regarding remanufactured products. As such, we investigated such public awareness in detail via a questionnaire survey, analyzed the extant problems and provided applicable suggestions. The data analysis of questionnaire survey was processed by cross-impact analysis and descriptive statistics with the help of SPSS software. 

### 3.1. Data Analysis of Questionnaire Survey

With constant efforts to collect data via the questionnaire survey over nearly five months, 611 valid questionnaires, with 87.3% response rate, were returned by the end of September 2017. In this section, descriptive statistics were applied to process the raw data and to accomplish further analyses. Results of data analysis follow.

• Basic knowledge of respondents on remanufactured products

Compared with the previous study that delivered questionnaires to officers in government and remanufacturers to investigate motives and barriers of remanufacturing [18], we intend to study the public awareness on remanufactured products from the perspective of consumers by allocating questionnaires in the Yangtze River Delta of China. Statistics demonstrated that 53.4% people of 611 valid respondents who took part in this survey were unaware of remanufactured products, compared with 46.6% people who heard and had basic understandings. The basic understandings related to remanufactured products include the concepts and definitions of the remanufactured products, differences between remanufacturing with renovation, distinction between original goods and remanufactured goods, etc. Overall, nearly half of the respondents had no idea about this new concept, indicating that remanufactured products have not been popular among Chinese residents so far. Hence, both the Chinese government and remanufacturing enterprises are faced with a difficult task of facilitating residents to thoroughly recognize the value of remanufactured products, as well as other similar environmentally-friendly goods.

Table 2 presents statistical results in the cognitive level of respondents to remanufactured products. Observations show that nearly 60% (57.6%) of male respondents had heard about remanufactured products. By contrast, the portion of female respondents was a little lower at 48.6%. Therefore, only about half of all respondents had heard of remanufactured products, suggesting that greater promotion of environmentally-friendly items is needed by government and enterprises. Outcomes of cognitive level recognition by gender showed that 9.0% more male respondent than female recognized remanufactured products, which can be explained by gender differences and distinctive attitudes toward commodities of construction machinery, electronic devices and automobile components, which comprise most remanufactured types. Specifically, compared with females, male respondents replied that they have more interest in remanufactured products, which focus on automobile components, electronic devices, etc. 

• Impacts of gender, age and education background on purchase intentions

Table 3 and Table 4 consider purchase experience and intentions of residents toward remanufactured commodities, both of which are correlated with gender, age and education background.

For the purchase experience in Table 3, 106 (32.8%) male respondents reflected that they have purchased some remanufactured products in their daily life, while the remaining 67.2% answered that they had never bought environmentally-friendly products such as remanufactured products. Female respondents responded almost the same as the male respondents.

Younger people (under 29 years old) have more experience in purchasing remanufactured products and have much stronger intentions to purchase these innovative products in the future. This phenomenon can be explained in two respects. In the first place, younger people are well versed in utilizing various information channels involving the Internet and other public media, facilitating their access to the remanufactured products and fostering their purchase intentions indirectly. Secondly, lower-income groups (respondents under 29 years old replied that most of them are still in the campuses as students without regular salary, or have part-time jobs with lower salary), compared with the populations of 30–39 years old and above, are more inclined to consume remanufactured products with competitive prices and guaranteed qualities. For the population whose ages are above 50 years old (50–59), results show that they have little experience in buying the remanufactured products, and they are reluctant to purchase them partly due to insufficient information from the Internet as well as the inadequate knowledge of environmentally-friendly commodities. From the conversations with them, respondents in this group replied that they have fewer chances to access to Internet compared with those in their twenties.

Although many respondents in the questionnaire survey turn out to lack the knowledge and cognition of remanufactured products, statistical results of purchase intentions appear to be promising. The majority of participants, 75.9% of male respondents and 68.4% of female respondents, expressed their willingness to purchase remanufactured products, based on payment-saving and environment-protection considerations. Combining the approximate 30% of respondents who have already purchased remanufactured products together with about 70% respondents who are willing to purchase in the future, it shows that both the potential of remanufactured products and the probable scale of the remanufacturing industry are enormous in China.

In Table 4, one can see that, compared with gender and age, the education background is less influential in customers’ purchase intentions. Based on the three respondent groups with different educational backgrounds (high school, bachelor and post graduates), about 70% of respondents in each group were glad to purchase remanufactured products, and approximately one-third of respondents in each group expressed that they have purchased remanufactured products before. Therefore, educational background is not a distinct impact factor in purchase intentions for Mainland Chinese nowadays.

• Knowledge and information channels of remanufactured products

**Figure 2:**
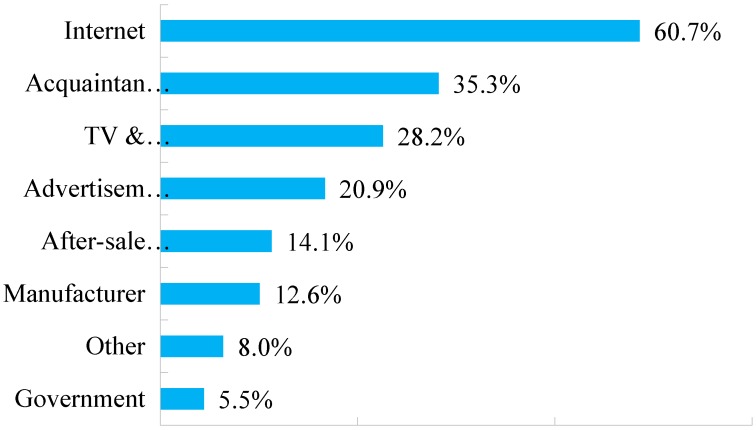
Information channels of remanufactured products.

Wang et al. [35] pinpointed that effective measures for promoting consumers’ purchase intention rely on multiple pillars such as product knowledge instead of single factors. From diverse information channels, customers would get product knowledge in varying degrees. When it comes to the knowledge and information channels of remanufactured products, a bar diagram (Figure 2) is shown to illustrate the data regarding channel distributions to disseminate remanufactured products. In response to information technology innovation and market expansion, the Chinese government has been promoting comprehensive development of the Internet by executing and invigorating strategies such as *Internet Plus* [52] to encourage enterprises to conduct online business. Due to this strategy, the Internet is becoming a pivotal channel for residents to acquaint themselves with remanufactured products—60.7% respondents agreed that they mainly turned to *Internet* for basic knowledge of remanufactured products. *Recommendation by acquaintances* was the response of 35.3% of the 326 respondents (see Figure 2) who have heard about remanufactured products from their friends and relatives, thus, informing a growing number of people of remanufactured products. The news of remanufactured products is increasingly expanding among residents, which has resulted in improved environmental awareness by the public.

*Television with broadcast* is the third most popular information channel for disseminating remanufactured products in this survey, comprising 28.2% of responses. Remaining items such as *Spread by advertisement on newspaper*, *Dissemination of after-sales service department* and *Propaganda of manufacturers*, occupied 20.9%, 14.1% and 12.6% of responses, respectively, a relatively small but important contribution to information for residents about remanufactured products. As previous surveys [19,34,53] have shown, enhanced recognition among residents of the remanufacturing industry and improved public awareness of environmental protection will be a logical sequel to remanufacturing progress, as statistical analyses in Figure 2 imply. 

• Diverse pre-purchase considerations of purchase intention

**Figure 3:**
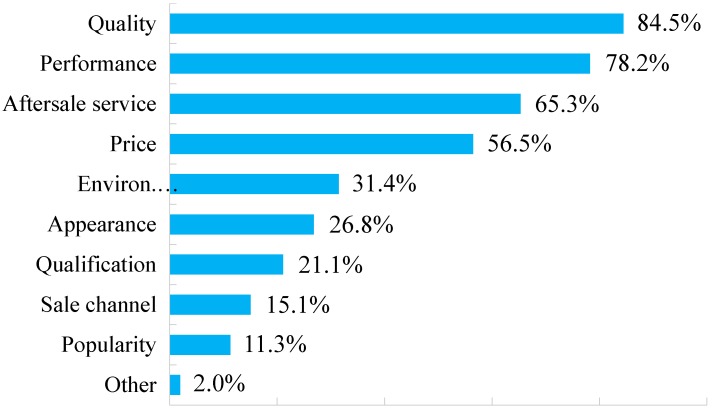
Pre-purchase considerations in purchasing remanufactured products.

Watson [33] found that price of remanufactured, repaired and reused products is the significant factor for customer purchase intention. Gaur et al. [54] utilized theoretical sampling technique to interview with consumers to research on drivers of consumer purchase intentions for remanufactured products, and found that environmental consciousness and social-cultural norms are the major drivers of consumer purchase intentions. However, via our survey, based on the perspective of remanufactured products themselves, we have some divergent results. Figure 3 shows the percentages of the pre-purchase considerations that customers care about when they purchase remanufactured products. The two most frequently answered considerations were the *Quality* and *Performance* of the products, arriving at 84.5% and 78.2%, respectively. Such high percentages indicate an unequivocal attitude of consumers towards the importance of the remanufactured product and its quality and performance. To alleviate public distrust in product attributes as well as to elevate purchase intention or willingness to purchase remanufactured products, *After-sale service* stands out as the third greatest consideration, comprising 65.3% of total respondents. Price is the fourth most important factor that customers focus on, at 56.5% of responses. Some respondents replied that they are willing to purchase the remanufactured products if the price is just 50–60% of the original ones, while the price ratio of 70–80% in reality appears to be less satisfactory. In addition, 205 respondents (26.8%) selected the item *Appearance*. Remanufactured products derived from used or scrapped products that are rusted, rotten or corrupted will not meet with customer satisfaction, but customers may be favorably impressed by the neat appearance of remanufactured commodities, stimulating their purchases as a result. 

The remaining two items of pre-purchase considerations, the *Sales channel* and *Qualification of manufacturers*, account for 21.1% and 15.1%, respectively. In our interviews with certain people, social popularity and remanufacturing competence of manufacturers are essential considerations during purchasing. The interviewees stated that they would trust the commodity quality if the products were remanufactured by some pilot enterprises such as SCMRC, WRG and XCMG possessing domestic certifications or provincial qualifications [55,56]. Besides, most customers show either a preference for the products being selected into *The List of Remanufactured Products* or remanufactured goods circulated in formal sales channels in the charge of reproducers or distributors with quality certifications, rather than unqualified private traders or second-hand stores.

• Purchase channels of remanufactured products

The Chinese government embarked on the promotion of the remanufacturing industry in 2013, via policy formulations and financial supports. Notwithstanding the engineering and technical strides made in remanufacturing industry, and notwithstanding the economic potential that remanufacturing presents, as the recent four years have shown, customer participation in remanufacturing must still be enhanced. In Table 3, 30.4% of respondents (186 of 611) have purchased remanufactured products before and, therefore, are aware of certain channels to access to them. The remaining 69.6% of respondents know little about these environmentally-friendly goods. 

**Figure 4:**
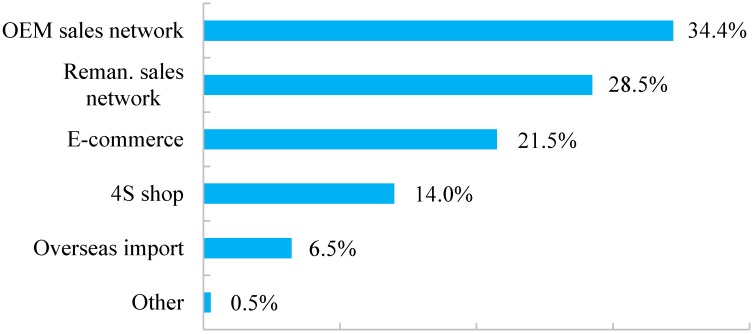
Purchase channels of remanufactured products.

The remanufactured commodities are generally reproduced and marketed by either the original manufacturers, or the third-party remanufacturers who are authorized to remanufacture certain brands of products. Hamzaoui-Essoussi et al. [29] suggested that OEM (Original Equipment Manufacturer) played an important role in remanufacturing/recycling strategy for enterprises and consumers. Such a finding is familiar to our statistical results. Among the 186 individuals, 34.4% responded that they were informed of remanufactured goods by *OEM* Sales network, as shown in Figure 4. *Sales network of the third-party remanufacturing enterprises* and *Electronic business platform* attracted 28.5% and 21.5% of the responses, and are partly due to advertising performances. The option *4S shop*, which refers to automobile sales service shops, occupied 14.0% since most consumers frequently visit such shops to disassemble broken or damaged parts and to replace them with new components, during which processes some remanufactured products are likely to be introduced to customers. Argo et al. [57] and Morales et al. [58] pointed out that consumers enjoy touching products themselves. Their studies explain why 14.0% respondents opted 4S shops for remanufactured products purchasing, where they are able to touch remanufactured products directly. It is evident from the data that, although some people are possessed with an elementary knowledge of purchase channels to access the remanufactured products, a comparatively smaller number of residents have such purchase experiences. Hence, it is imperative for both the government and remanufacturing enterprises to enlarge existing channels for the promotion of remanufactured products among potential customers. 

• Responsible entities for promoting remanufactured products

**Figure 5:**
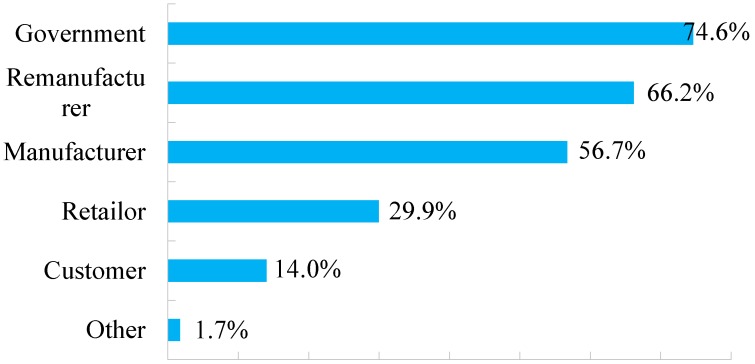
Responsible entities for promoting remanufactured products.

Respondents whose intentions were more aligned with their moral norms (such as awareness of energy conservation, environmental consciousness and attitude to emission-reduction) are more likely to perform specific behaviors [59]. With the growth of environmental consciousness, responsible entity for promoting remanufactured products is becoming an essential factor that all parties (enterprises and government) should consider to promote remanufactured goods. Overall, 94.3% of respondents consider it essential to popularize the remanufactured products for environmental protection. As to the responsibility for the dissemination processes, 74.6% respondents in Figure 5 held the idea that the *Chinese government* should bear the prime obligation as the social planner. Supporting measures and productive incentives should be embraced for the efficacious propaganda of remanufacturing superiority in economic and environmental progress. 

The second largest proportion, *Remanufacturing enterprises*, accounted for 66.2% and was followed by *Retailers* and *Customers*, at 29.9% and 14.0%, respectively. Hazen et al. [60] pointed out that a firm’s adoption of green reverse logistics leads to higher levels of consumer loyalty. It can explain the phenomenon that more than half of respondents think that *Remanufacturing enterprises* and *Retailers* should shoulder the responsibility of promoting remanufactured products. After all, these enterprises can adopt green reverse logistics and win consumer loyalty. Overall, the nation, corporations and residents are supposed to work jointly on the dissemination and promotion of remanufactured goods.

• Propaganda paths of remanufactured products in China

The diverse measures in Table 5 on popularizing the remanufactured products in China make it clear that 83.1% respondents advocated for the government to set-up Quality Assurance Systems and to enhance legislation in the realm of remanufacturing and circular economy. The State Council has established technology and quality testing centers for remanufacturing at a national level, through the enactment and implementation of *Recommendations on Promoting Remanufacturing Industry* issued in August 2008. The National Quality Testing Center, however, fails to match quality audit requirements at distinctive standards over the country; hence, local authorities are encouraged to set up district testing centers for quality inspections of the remanufactured products. 

Overall, 44.1% of respondents deemed propaganda in campuses and communities an insightful approach to further popularize remanufactured products in China, considering students and teenagers as potential great beneficiaries. Approximately 40% people reflected that the subsidies on remanufactured products from the government could intensify the dissemination, because such financial support directly leads to increased revenues of remanufacturing and to competitive pricing in the market. In total, 31.4% respondents stated that the government should increase the procurement of remanufactured products. Once the local governments buy more remanufactured products, residents are more likely to initiate purchasing. In total, 30.9% respondents expressed their willingness to share the purchase experiences with their acquaintances if the qualities and performances of remanufactured products are satisfactory.

### 3.2. Interview Results 

The interviews with the respondents whose answers were recorded provided Appendix A to verity the outcomes from questionnaires. For instance, when respondents replied to the questions “Have you ever heard of remanufactured products?” and “Do you know the concepts of it?”, they replied that they feel ambiguous about remanufactured products and cannot easily distinguish “remanufacturing” from “renovation”. 

When it comes to the question “Which attribute of remanufactured products do you care most? For example: price, capacity etc.”, respondents reflected that comparing with price, they care about capacities of these kinds of products most, because the price of remanufactured products is much lower than original ones and they can afford this price. The outcomes of interviews are coincident with the results from questionnaire: In Figure 3, Price is the fourth important factor which is behind Quality, Performance and After-sale service.

For the responsible entity of environmental protection, almost all interviewees said that government, remanufacturer and manufacturer should undertake joint responsibilities to create an environmentally-friendly society. Beside governments and enterprises, respondents also agreed that customers should also protect environment positively and cooperate with other parties to make a concerted effort. 

Our interviews were conducted in various public places such as campus, squares workshops and automobile-part shops. In order to understand the public awareness of remanufactured products further, the interviewees who we selected have different occupations such as students, faculties of campus, workers and salesmen. All the questions in the interview were answered by both male and female respondents. Besides, the arrange of interviewer age covered from under 20 years old to 60 years old above. Based on their answers, we finally summarized a series of viewpoints of the interviewers. Some authentic quotations of respondents in recorded interviews are shown in Table 6.

## 4. Problems and Recommendations

Public awareness of Chinese residents of remanufactured products has strengthened during the past several years but is still insufficient. This emerging product is fostered by related policies and regulations such as *Swap the Old for Remanufacturing* in China currently, and draws more investment and attention from enterprises. According to our investigations and analyses, however, there remain problems to be solved from the perspectives of government and corporations. The latent problems are discussed in this section and corresponding recommendations are proposed for the further promotion of remanufactured products.

• Weak awareness and lack of propaganda on remanufactured products

Overall, 46.6% respondents replied that they never heard about the remanufactured products (Table 2). As for the related policies such as *Swap the old for remanufacturing*, *Sample pilot enterprises of remanufacturing* and *The List of Remanufactured Products*, only 29.3% respondents were aware of such regulations. It is obvious that public recognition of remanufactured products and other environmentally-friendly goods is comparatively weak in China. Moreover, relevant publicity of remanufactured products is far from enough; the policies and legislation in the field of remanufacturing have aroused only slight public attention and often lag in execution.

Faced with these challenges, applicable and prudent measures should be taken at both authority and industry levels. From the perspective of the government, expanding the channels of information disclosure is supposed to be accelerated. For instance, central government can record and broadcast public service advertising to emphasize the environmental benefits of the remanufactured products. According to Figure 2, 28.2% of respondents who are conscious of remanufactured products become acquainted with it via TV and broadcast, thus the broadcasting of public service advertising is essential to get residents familiar with environment-favored goods. The Internet should be utilized to unmask detailed information, including certain kinds of remanufactured commodities mentioned in *The List of Remanufactured Products*. By searching the categories or types of remanufactured products online, potential buyers are likely to be spurred to make purchases. Residents can also have a sound understanding of remanufacturing via the Internet, along with its products. 

From the perspective of remanufacturing enterprises, newspaper and electronic business platforms are useful options to introduce various remanufactured products to the public. Remanufacturing enterprises can advertise their products in the provincial or local newspaper to disseminate basic knowledge, and then make the most of e-business. Sinnorc.com, one of the emerging popular trading websites, which was set up in 2014, now has turned into the largest electronic business platform of remanufactured auto parts in China. The transaction amount in Sinnorc.com has exceeded 20 million RMB (~$3.03 million) by the end of 2017, and nearly 500 remanufacturing enterprises have founded online shops and distributors to exhibit and sell products on the platform. Among these, remanufactured auto parts are presented which mainly refer to engines, alternators and transmissions, including about 100 brands such as Mercedes Benz, Audi and Toyota. Remanufacturing enterprises can utilize such platforms to enlarge their channels for spreading more information, and to exhibit their environment-preferred products to potential customers.

• Deficient legislation on remanufacturing and lacking dissemination of policies

For the sake of promoting the remanufacturing industry along with its products, enactment of laws is regarded as one indispensable measure. In practice, Chinese government published *The Circular Economy Promotion Law* in August 2008, planning to facilitate and regulate remanufacturing in car parts, machinery, etc., which requires all products meet national quality standards, and be marked clearly with remanufacturing labels. While such laws and regulations have been issued and have indeed worked to the advancement of remanufacturing industry and remanufactured products, they are generally legislated at a comprehensive level, applying to various trades and professions covering remanufacturing and related occupations. In this sense, specific laws targeting the remanufacturing industry have been absent so far. In accordance with reality, our statistical results show that 70.7% respondents are unaware of policies such as the *Swap the Old for Remanufacturing* which is the most noticeable legislation regarding remanufacturing, published in 2013. It stipulated that subsidies should be provided for remanufacturing enterprises from the government, and customers are entitled to buy remanufactured products at some discount, 15% on average, if used products of the same kind are collected. Existing supporting legislation is suggested to be promulgated and popularized for a better understanding of improved customer benefits.

For further promotion of remanufactured products, legislation by authority is indispensable and imminent. When the remanufacturing industry is included in the regulation and surveillance of specific laws and rules, enterprises can produce remanufactured products and process end-of-life products in conformity with legal provisions. Customers, on the other hand, are entitled to protest their rights according to the law, when suffering from undesirable purchasing of defective products. Once improved policies on remanufacturing prove to be applicable, corresponding dissemination channels should be enhanced. Chinese government has deployed policies and regulations on the official websites of MIIT, the State Council, etc., where residents have little chance to visit and consider them, unrelated to their daily life. Therefore, the government and enterprises are instead advised to strengthen the mass media such as micro-blog, WeChat (the most popular instant communication application in China) and to recourse to campus campaigns. Moreover, specific news release conferences are essential to introduce recent laws and regulations on the remanufacturing industry, at which time journalists will spread up-to-date policies attracting public attentions.

• Poor sales performance and weak enthusiasm of customers

Based on our research results, about one-third (30.4%, 186 of 611) of respondents reflected that they have access to remanufactured products. Most of them, 34.4% and 28.5% of the 186 respondents, stated that they purchased the remanufactured products via original manufacturers and from third-party remanufacturing enterprises (Figure 4). Statistical results are evident that the majority of remanufactured products in current China are collected, scrapped and marketed by original manufacturers, manifesting as an OEM remanufacturing mode. Third-party enterprises of remanufacturing are sometimes allowed to process and distribute remanufactured products after authorization, also generating a considerable number of remanufactured products. According to Table 4, 72.3% respondents expressed their willingness to buy remanufactured products if the price is 50–60% of the original price, clarifying current pricing of remanufactured commodities with a 20–30% reduction of newly-made ones as an undesirable outcome, and discouraging potential customers to make purchases at that price.

To enrich sales channels and improve the purchase intentions of customers, advice for firms regarding original manufacturers and third-party remanufacturers are provided as follows. 

1. Original manufactures are suggested to distribute diverse remanufactured products such as engines, electric generators and starters in their 4S shops. 

Since customers in need of repairing and replacing auto components visit 4S shops frequently, salesmen could promote remanufactured commodities along with their preferential attributes. Customers are therefore able to compare the properties and retail prices between the remanufactured goods and original ones more directly, recognizing advantages of remanufactured products and facilitating rational purchasing in some respects. 

2. Original manufacturers can encourage customers to swap the scrapped parts for discounts when purchasing remanufactured products. 

Since scrapped parts are major materials for reproducing, customers can be offered 10–20% discounts when buying remanufactured products if the used or end-of-life parts are collected. By implementing this measure, time- and cost-saving targets of recycling scrapped parts for manufacturers are attained, as well as purchase intentions of customers on remanufactured products enhanced.

3. Online trading of remanufacturing should be invigorated and further promoted in the *Internet Plus* setting. 

Remanufacturing enterprises can build up their own online shops in T-Mall (the largest online shopping mall in China) to sell remanufactured products. Customers participate by trading used or scrapped parts to offset a portion of payments on online shopping, and the remanufactured commodities are then sent to them by the third-party logistics. Joint contribution of remanufacturing enterprises, customers and third-party logistics service providers can make up a closed supply chain in which convention cost and time between the remanufactured commodities and waste products can be significantly economized.

• Lack of quality standards and passive quality inspections

The quality of the remanufactured products is a prior consideration for customers when they decide whether to make purchases. It can be seen in Figure 2 that 84.5% and 78.2% respondents reflected their worries about the quality and performance of the remanufactured products, doubting whether the remanufactured products can meet their requirements like the original products do. Approximately 70% (69.7%) of respondents replied that the remanufactured products made by the third-party remanufacturing firms with the domestic certification would be trusted.

In the light of the statistical results, creating uniform standards of quality and enhancing quality inspection will be a benefit to the sales of the remanufactured commodities and other environmentally-friendly products. Related suggestions are put forward as follows. 

1. The government is advised to invest more on research of quality standards and to implement those standards. 

Total types of the remanufactured products in China have exceeded 10,000, while comparatively less uniform standards of only 48 kinds have been published so far. Thus, formulating targeted uniform standards for certain types of remanufactured products becomes an urgent assignment for the government to fulfill. Specifically, investigations on quality specifications of pilot remanufacturing enterprises should be underscored during the deliberation process of product quality standards, through which more reasonable standards are likely to be addressed for comprehensive adoption.

2. Establishment of national quality testing institutes will help governments to inspect and test quality, with more branches to be founded in various districts. 

Inspired by the publication of *Recommendations on Promoting Remanufacturing Industry* in 2008, the national quality testing center was then set up two years later in Beijing, China, for quality inspection of remanufactured products nationally. To test the remanufactured products in conformity with specific standards, local testing centers are essential.

3. The government can publish the uniform standards via various media such as newspapers, television and the Internet. 

Due to the publicity of the uniform standards, customers can distinguish qualified remanufactured products from inferior ones. If the remanufacturing enterprises produce unqualified products, they will be exposed and fined, and exemplary firms will be removed from the list of sample pilot remanufacturing enterprises.

## 5. Conclusions, Limitations and Future Research

This paper investigates the public awareness of Chinese residents for remanufactured products by questionnaire survey, field research and interview, information search and literature study, especially by collecting and analyzing data from 611 valid questionnaires. Generally, public awareness on emerging products with environment-favorable attributes was relatively weak. Relatively few residents are possessed with a sound knowledge of remanufactured products due to deficient information from governments and enterprises. Moreover, nearly 70% of them have not purchased remanufactured products despite 72.3% of respondents who express their willingness to pay. Fortunately, most respondents believe it will be necessary and important to popularize environmentally-friendly products, and both the government and enterprises are supposed to be in charge of the duty to protect and ameliorate environment pollution [61,62]. 

With regard to the extant problems, related suggestions for the government and enterprises are expounded. Both authorities and firms should be aware that the public recognition and preference for remanufactured products will influence their purchasing considerably. Therefore, remanufactured products should be promoted via various kinds of media, with the help of formulating targeted quality standards, inspection mechanisms, etc. Although improvements are needed, the remanufacturing industry and its products will contribute to the economy and to quality of life of residents, while benefiting environment protection. 

We consider the paper of significant contributions in theory and method.

- Descriptive survey and cross-impact analysis methods were utilized to collect and process raw materials and data. Both methods are distinguishable from previous empirical studies and mathematical modeling that adopted scaling method to carry out factor analysis in remanufacturing.
- Although questionnaire surveys of Chinese remanufacturing industry have been conducted previously, their respondents are mainly staff of enterprises and officers of governmental organizations. We targeted Chinese residents since consumers are the main driver to prompt this innovative industry.

In China, regions such as Pearl River Delta and Beijing-Tianjin-Hebei urban agglomeration whose economic development, industrialization and urbanization degrees are similar to Yangtze River Delta. Hence, this paper provides valuable suggestions, from a practical perspective, for the local authorities to popularize remanufactured products all around the nation. For other countries with similar status of remanufacturing industry, India and Malaysia for instance, our work can also provide sensible and applicable implications to promote remanufactured commodities. 

Although the current paper along with its results has crucial significance from both theoretical and pragmatic aspects, it still has several limitations that merit future research.

- By considering various variables, empirical research methods such as framework building can increase the relevance of the research further. Scaling method, for instance, is worth studying.
- Since remanufacturing in China is currently concentrated in Yangtze River Delta, we did not distribute questionnaires in west and north areas where density of population is low, and finance and human resources are limited. However, considering unremitting progress of Chinese remanufacturing industry in the future, we believe promotions and public awareness of remanufactured products in these areas are worth investigating.

## Figures and Tables

**Table 1:** Demographic results of questionnaire survey.

**Questionnaire Numbers**	**Age**					
	<20	20–29	30–39	40–49	50–59	≥60
	81 (13.3%)	251 (41.1%)	108 (17.7%)	135 (22.1%)	30 (4.9%)	6 (1%)

**Table 2:** Cognitive level of remanufactured products.

Cognitive Level	Gender		Age						Education Background		
	Male	Female	Under 20	20–29	30–39	40–49	50–59	Above 60	High School	Bachelor	Post Graduates
Heard	186 (57.6%)	140 (48.6%)	43 (53.1%)	145 (57.8%)	53 (49.1%)	71 (52.6%)	13 (43.3%)	1 (16.7%)	111 (52.4%)	136 (49.6%)	79 (63.2%)
Not heard	137 (42.4%)	148 (51.4%)	38 (46.9%)	106 (42.2%)	55 (50.9%)	64 (47.4%)	17 (56.7%)	5 (83.3%)	101 (47.6%)	138 (50.4%)	46 (36.8%)

**Table 3:** Purchase experience of residents for remanufactured products.

Purchase Experience	Gender		Age						Education Background		
	Male	Female	Under 20	20–29	30–39	40–49	50–59	Above 60	High School	Bachelor	Post Graduates
Have purchased	106 (32.8%)	80 (27.8%)	30 (37.0%)	78 (31.1%)	32 (29.6%)	38 (28.2%)	8 (26.7%)	0 (0.00%)	63 (29.7%)	88 (32.1%)	35 (28.0%)
Have not purchased	217 (67.2%)	208 (72.2%)	51 (62.9%)	173 (68.9%)	76 (70.4%)	97 (71.9%)	22 (73.3%)	6 (100.0%)	149 (70.3%)	186 (67.9%)	90 (72.0%)

**Table 4:** Purchase intentions of residents on remanufactured products.

Purchase Intentions	Gender		Age						Education Background		
	Male	Female	Under 20	20–29	30–39	40–49	50–59	Above 60	High School	Bachelor	Post Graduates
Willing	245 (75.9%)	197 (68.4%)	52 (64.2%)	203 (80.9%)	67 (62.0%)	101 (74.8%)	18 (60.0%)	1 (16.7%)	144 (67.9%)	212 (77.4%)	86 (68.8%)
Not willing	78 (24.2%)	91 (31.6%)	29 (35.8%)	48 (19.1%)	41 (38.0%)	97 (71.9%)	12 (40.0%)	5 (83.3%)	68 (32.1%)	62 (22.6%)	39 (31.2%)

**Table 5:** Measures to propagandize remanufactured products.

Items	Counts	Percentages (%)
Government set up Quality Assurance System and legislate on remanufacturing industry	508	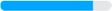 83.1
Propaganda in schools, communities etc. by the government	269	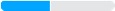 44.1
Government subsidizes on remanufactured products	239	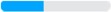 39.1
Advertisements and sales promotion by remanufacturers and suppliers	237	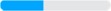 38.8
Increase government procurement of the remanufactured products	192	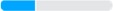 31.4
Customers propagandize purchase experiences to their acquaintances	185	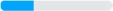 30.9
Others	24	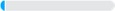 3.9

**Table 6:** Quotations of questions and answers in interviews.

Questions	Answers
*Have you ever heard of remanufactured products? Do you know the concepts of them?*	*I heard about remanufactured products but cannot clearly distinguish them between renovated products such as renovated machines.*
*Which kind of information channels help you to understand remanufactured products?* *Have you known where you can purchase the remanufactured products?*	*Internet is the major channel for us to understand emerging products, besides, advertising in TV & broadcast also provides related information of remanufactured products to us. Moreover, our friends who have bought them in the 4S shops sometimes will recommend the products to me.*
*Which attributes of remanufactured products do you care most? For example: price, capacity, etc.*	*As far as I know, the retail price of remanufactured products is about 50–60% of the original ones which I can afford. Hence, we regard capacity and performance as the prime concerns.*
*Have you ever heard some national policies such as the “Swap the Old for Remanufacturing”?* *Do you think it is necessary for government and enterprises to propagate remanufactured products?*	*Actually, I did. But, I did not clearly know the objectives or benefits of this proposal. It is very necessary to popularize remanufactured products due to increasingly sever environmental problems.*
*Do you agree with the opinion that “it is important to protect environment nowadays”?* *Who should undertake the responsibility of environmental protection?*	*First, it must be government who have the power to publish regulations to protect environment. Then, manufacturers also have to do environmental protection because they produce the products. Customers also should undertake part of the responsibility of environmental protection.*

## Appendix A

**Survey about Public Awareness on Chinese Remanufacturing Industry**

**Related Definitions in the Questionnaire**

***Remanufacturing***—The process of collecting used parts or products, bring them to as-new conditions by procedures such as disassembling, cleaning, quality check, repairing and replacing parts, reassembling, testing, etc.

***Original Manufacturer/Remanufacturer***—Enterprises that remanufacture products of its own brand.

***Third-party Remanufacturer***—Enterprise that is possessed with national licenses of NDRC (National Development and Reform Commission) and remanufactures products with the brands of other firms.

***Questionnaire***

1. Your Gender
  ○ Male
  ○ Female
2. Your Age
  ○ below 20 ○ 20–29 ○ 30–39
  ○ 40–49 ○ 50–59 ○ above 60
3. Which city do you live in now?
  _________________________________
4. Your monthly income in yuan
  ○ Below 1500
  ○ 1500–5000
  ○ 5000–10,000
  ○ 10,000–15,000
  ○ Above 15,000
5. What’s your education level?
  ○ Primary school and junior high school
  ○ Senior high school
  ○ Undergraduates
  ○ Postgraduates
6. Your vocation
  ○ Students ○ Service ○ Agriculture
  ○ Industry ○ Business ○ Civil Servant
  ○ Retired ○ Other
7. Have you ever purchased remanufactured products?
  ○ Yes ○ No
8. Have you ever heard about remanufactured products?
  ○ Yes
  ○ No (Skip to 11th)
9. Which channels help you know about remanufactured products?
  □ Friends and Relatives □ Manufacturer □ Retailer
  □ Internet □ Advertisement □ Government policy and regulation
  □ Television and Broadcast □ Other _________________
10. Which channels that promulgate the remanufactured products do you trust most?
  □ Government □ Original Manufacturer □ Retailers and 4S shops
  □ Remanufacturing enterprises □ Maintenance Stations □ Specialist
  □ Non-government Organization □ Friends and Relatives □ Other _________________
11. Are you willing to purchase the remanufactured products if the price of them are only 50–60% of the new products?
  ○ Yes (Skip to 13th)
  ○ No
12. Which factors inhibits you to purchase remanufactured products?
  □ The qualities of remanufactured products are not as same as the new products
  □ The safety of remanufactured products
  □ The economic expense in remanufactured products
  □ The after-sale service of remanufactured products
  □ Retailer use low-quality products to replace remanufactured products
  □ Other _________________
13. Which channels of remanufacturing do you trust?
  □ Original manufacturers produce remanufactured products
  □ Third parties who get the national license of remanufacturing
  □ Maintenance Station
  □ Manufacturers of the same type products
  □ Other _________________
14. In which respects do you care most about the remanufactured products?
  □ Appearance □ Capacity □ Quality
  □ Price □ After-sale service □ Sale Channel
  □ Popularity □ Qualification of remanufacturing enterprises □ Environmental
  □ Other _________________
15. Do you know how to purchase remanufactured products in some formal channels?
  ○ Yes
  ○ No (Skip to 17th)
16. Do you know where you can purchase the remanufactured products?
  □ Sale network of original manufacturer
  □ Supermarket
  □ E-commerce
  □ Sale network of the remanufacturing enterprise
  □ Overseas Import
  □ Other _________________
17. Have you ever noticed any propaganda of remanufacturing?
  ○ Yes
  ○ No
18. Have you ever heard about remanufacturing policies such as “Swap the old for Remanufacturing”?
  ○ Yes
  ○ No
19. Do you think it is necessary to propagate remanufacturing?
  ○ Yes
  ○ No
20. Who do you think should be responsible for the popularizing the remanufactured products?
  □ Government
  □ Manufacturer
  □ Remanufacturing enterprises
  □ Retailer
  □ Customer
  □ Other _________________
21. Which ways do you think facilitating the development of remanufacturing industry?
  □ Laws and regulations to facilitate remanufacturing industry
  □ Propaganda of remanufacturing in campuses, communities etc.
  □ Government procurement
  □ Subsidy on remanufactured products
  □ Advertisement about remanufacturing
  □ Customers introduce remanufactured products to their friends and relatives
  □ Other _________________
